# Quantitative Profiling of Carotenoids, Tocopherols, Phytosterols, and Fatty Acids in the Flower Petals of Ten Marigold (*Tagetes* spp. L.) Cultivars

**DOI:** 10.3390/foods12193549

**Published:** 2023-09-24

**Authors:** Ramesh Kumar Saini, Hui-Yeon Ahn, Geon-Woo Park, Ji-Won Shin, Jung-Hoon Lee, Ji-Woo Yu, Min-Ho Song, Young-Soo Keum, Ji-Ho Lee

**Affiliations:** Department of Crop Science, Konkuk University, Seoul 143-701, Republic of Korea; saini1997@konkuk.ac.kr (R.K.S.); gmldus4697@konkuk.ac.kr (H.-Y.A.); pgw0111@konkuk.ac.kr (G.-W.P.); jiwonee02@konkuk.ac.kr (J.-W.S.); elee4128@naver.com (J.-H.L.); wooody96@konkuk.ac.kr (J.-W.Y.); hlhkkl@konkuk.ac.kr (M.-H.S.); rational@konkuk.ac.kr (Y.-S.K.)

**Keywords:** lutein, α-tocopherol, lutein-myristate-palmitate, lutein dipalmitate, ABTS^•+^, DPPH^•^, *Tagetes patula* L., *Tagetes erecta* L., β-sitosterol

## Abstract

Marigold (*Tagetes* spp.) flower petals are the most vital sources of carotenoids, especially lutein esters, for the production of natural lutein to use for food, feed, and pharmaceutical industries. Several marigold cultivars are cultivated globally; however, their lutein ester composition and contents have not been widely investigated. Considering this, this study aimed to identify and quantify prominent carotenoid esters from the flower petals of ten marigold cultivars by liquid chromatography (LC)–diode-array detection (DAD)–mass spectrometry (MS). In addition, tocopherols, phytosterols, and fatty acids were analyzed by gas chromatography (GC)–flame ionization detection (FID) and GC–MS. Furthermore, the 2,2′-azino-bis(3-ethylbenzothiazoline-6-sulfonic acid (ABTS^•+^) and 2,2-diphenyl-1-picrylhydrazyl (DPPH^•^) radical scavenging abilities of lipophilic extracts were determined. The total carotenoid contents varied significantly (*p* < 0. 05, Tukey HSD) among cultivars, ranging from 25.62 (*cv.* Alaska)–2723.11 µg/g fresh weight (*cv.* Superboy Orange). Among the five major lutein-diesters, (all-*E*)-lutein-3-*O*-myristate-3′-O-palmitate and lutein dipalmitate were predominant. Among the studied cultivars, α-tocopherol was recorded, ranging from 167.91 (*cv.* Superboy Yellow) to 338.50 µg/g FW (*cv.* Taishan Orange). Among phytosterols, β-sitosterol was the most prevalent phytosterol, ranging between 127.08 (*cv*. Superboy Yellow) and 191.99 µg/g FW (*cv*. Taishan Yellow). Palmitic acid (C_16:0_; 33.36–47.43%) was the most dominant among the fatty acids. In this study, the highest contents of lutein were recorded from *cv.* Superboy Orange; however, due to the substantially higher flower petal yield, the *cv.* Durango Red can produce the highest lutein yield of 94.45 kg/ha. These observations suggest that *cv.* Durango Red and *cv.* Superboy Orange are the ideal candidates for lutein fortification in foods and also for commercial lutein extraction.

## 1. Introduction

All terrestrial and aquatic photoautotrophs, such as plants, microalgae, and macroalgae, universally produce carotenoids, the most prevalent class of tetraterpenoid pigments. Carotenoids can be categorized into two groups: (i) carotenes, which consist of a parent hydrocarbon chain without any functional group, such as β-carotene, and (ii) xanthophylls, which have oxygen as a functional group, exemplified by lutein and zeaxanthin. Carotenoids play a vital role in human health due to their antioxidant and provitamin A activities [1,2,3].

Humans accumulate xanthophylls, namely lutein and zeaxanthin, as a macular pigment in the retina’s fovea and inner plexiform layer. These pigments play a crucial role in safeguarding the retinal membrane from the detrimental effects of short-wavelength high-intensity light and enhance visual acuity [4,5]. Moreover, clinical and epidemiological studies have observed the critical role of dietary lutein and zeaxanthin in cognitive improvement [6,7,8], reducing the risk of age-related macular degeneration (AMD) [5], age-related declines in muscle mass and strength [9], cardiometabolic health [10], and reducing the risk of cancer [11,12].

Colorful fruits and vegetables constitute the primary dietary source of carotenoids in the human diet [13]. However, in recent years, microalgae, macroalgae, and food waste have also been explored to extract natural carotenoids [14,15,16,17,18,19]. Among the natural sources, marigold (*Tagetes patula* L. and *T. erecta* L.) flower petals are the most vital sources of carotenoids, especially lutein diesters [20]. Considering the high content of lutein, the industry extensively uses marigold flowers for natural lutein production for functional food, feed, cosmetics, and pharmaceuticals [19]. In addition to lutein extraction, marigold flowers are extensively used in poultry feed to improve egg yolk pigmentation, a symbol of high nutritional value and marketing quality eggs [21,22].

In marigold flower petals, lutein acylated with saturated fatty acids, such as stearic (C_18:0_), palmitic (C_16:0_), myristic (C_14:0_), and lauric (C_12:0_) acid moieties, are dominantly found [23]. Interestingly, these fatty acids are also associated with other xanthophylls found in different plants [24]. When the identical fatty acid is esterified on both sides of the lutein molecule, it results in a lutein homodiester (e.g., lutein-dimyristate). On the other hand, esterifying lutein with two different types of fatty acids forms a heterodiester (e.g., lutein-3-*O*-myristate-3′-*O*-palmitate) [25]. Interestingly, as lutein is an asymmetric molecule due to the presence of a β-ionone ring at one side and an ε-ionone ring on another side, esterification with two different kinds of fatty acids gives rise to lutein ester regioisomers—for example, lutein-3*-O-*palmitate-3′*-O-*myristate and lutein-3-*O*-myristate-3′-*O*-palmitate.

Several studies have attempted to quantify the lutein contents in marigold flower petals. However, most of these studies quantified the lutein contents in saponified samples [26,27,28]. In saponification, fatty acid moieties attached to the carotenoid molecules are removed, yielding free carotenoids. This process makes quantification easy; however, this approach does not provide information about naturally occurring carotenoid esters. In recent years, the availability of advanced liquid chromatography (LC)–mass spectrometry (MS) techniques means that it is possible to separate and identify the complex carotenoid esters regioisomers in the native samples [29,30].

Apart from carotenoids, phytosterols represent another class of beneficial compounds that help to regulate low-density lipoprotein (LDL) cholesterol levels and endothelial function [31]. The US Food and Drug Administration (FDA) recommends a daily dietary intake of 2 g of non-esterified plant sterols to achieve the health advantages of reducing blood total and LDL cholesterol and lowering the risk of coronary heart disease (CHD) [32].

Tocopherols (α-, β-, γ-, and δ; commonly known as vitamin E) are essential constituents of cellular lipids. They play a crucial role as a chain-breaking antioxidant, interrupting and mitigating the propagation of harmful oxidative reactions that can lead to cellular damage and degradation of lipids, proteins, and DNA [33]. Consequently, this aids in reducing the susceptibility to diseases linked with oxidative stress, encompassing conditions such as cardiovascular diseases, neurodegenerative diseases, various types of cancer, and cognitive deterioration linked to aging [34,35,36,37]. Moreover, α-tocopherol is believed to improve skin health and immune function [38].

Considering the commercial importance of lutein obtained from marigold flower petals, the objective of this work was to carry out an identification of prominent carotenoid esters from the flower petals of ten marigold cultivars by LC-diode-array detection (DAD)-MS, followed by their quantification by LC-DAD. In addition, tocopherols, phytosterols, and fatty acids were analyzed by gas chromatography (GC)–flame ionization detection (FID) and GC–MS methods. Furthermore, the 2,2′-azino-bis(3-ethylbenzothiazoline-6-sulfonic acid (ABTS^•+^) and 2,2-diphenyl-1-picrylhydrazyl (DPPH^•^) radical scavenging abilities of lipophilic extract were determined. The lutein contents of marigold cultivars commonly cultivated in Korea can help to recommend the lutein-rich and high flower-yielding cultivars for the industrial extraction of lutein and functional food preparations.

## 2. Materials and Methods

### 2.1. Reagents, Standards, and Plant Material

Seeds of four Aztec (*T. erecta* L.) and six French (*T. patula* L.) cultivars were obtained from Asia Seeds Co., Ltd., Seoul, Korea (Table 1). The seeds were cultivated in the greenhouse under prevailing humidity and temperature conditions. The mean temperature was 15 °C during seed showing (April 2022) and 30 °C during flower harvesting (June 2022). Four pots, each measuring 28 × 48 cm, were used to grow every cultivar. These pots were filled with a commercial potting mixture supplied by Asia Seeds Co., Ltd., Seoul, Korea. In each pot, six plants were maintained. The plants were cultivated without applying any pesticides and fertilizers. The fully developed flowers were harvested on several occasions till the end of flowering. The flowers were cleaned, the petals were separated, the total weight was recorded, and the samples were stored in a −80 °C deep freezer, until analysis.

Authentic standards of (all-*E*)-β-carotene, (all-*E*)-lutein, β-sitosterol (24α-ethyl cholesterol), campesterol (24α-methyl cholesterol), 5-β-cholestan-3α-ol (utilized as an internal standard), fatty acid standard mix (CRALASKA7885), 2,2-diphenyl-1-picrylhydrazyl (DPPH^•^), 2,2′-azino-bis(3-ethylbenzothiazoline-6-sulfonic acid) (ABTS^•+^), and 6-hydroxy-2,5,7,8-tetramethylchroman-2-carboxylic acid (Trolox) were obtained from Merck Ltd., Seoul, South Korea. ChromaDex, Inc., located in Irvine, CA, USA, supplied the tocols mix solution, which includes α-, β-, γ-, and δ-tocotrienol, and α-, β-, γ-, and δ-tocopherol. The (all-*E*)-violaxanthin utilized in this study was purified from lettuce following our established protocol [39].

The organic solvents used for extraction and analysis were of LC grade and were provided by J.T. Baker^®^, Suwon-Si, Korea.

### 2.2. Extraction of Carotenoids, Sterols, and Fatty Acids

The major lipophilic compounds comprising carotenoids, tocopherols, phytosterols, and fatty acids were extracted (under low light conditions) from fresh marigold flower petals following our optimized protocol [40] with slight adjustments. The comprehensive extraction procedure is provided in Supplementary Figure S1.

A portion of the extracted sample (0.5 mL) was subjected to hydrolysis and transformed into fatty acid methyl esters (FAMEs) utilizing the methanolic hydrochloric acid (HCl) reagent, as outlined in Figure S2. Similarly, trimethylsiloxy [−O-Si(CH_3_)_3_; TMS] derivatives were prepared for phytosterol analysis, as outlined in Figure S3 [41].

### 2.3. LC-MS Analysis of Carotenoids and Tocopherols

The prominent carotenoids and tocopherols in the lipophilic extract were identified and quantified utilizing the LC-photodiode array (PDA)–mass spectrometry (MS) method. The LC (Shimadzu, Tokyo, Japan) was equipped with an LCMS-9030 quadrupole time-of-flight (Q-TOF) mass spectrometer (Shimadzu, Tokyo, Japan) and an SPD-M20A PDA detector (Shimadzu, Tokyo, Japan).

The LC-PDA-MS parameters are summarized in Table 2. The carotenoids and tocopherols were identified by co-chromatography with authentic standards, UV–VIS spectral features, mass spectra characteristics, and comparison with literature data [29].

The identified free and esterified carotenoids were quantified using five-point external calibration curves (5–50 µg/mL) of respective non-esterified carotenoid standards obtained by LC-PDA. Carotenoid esters were estimated using the curve of the corresponding free carotenoid, as attachment of fatty acids does not significantly influence the PDA spectra. Similarly, tocopherols were quantified using five-point external calibration curves (10–100 µg/mL) of respective standards.

### 2.4. Fatty Acids Analysis

The qualitative analysis of FAMEs was conducted using GC-FID (Agilent 7890B, Agilent Technologies Canada, Inc., Mississauga, ON, Canada). The optimized instrumental conditions are summarized in Table 3 [43]. In order to ensure the accurate identification of FAMEs, the GC-mass spectrum of representative samples was recorded using the QP2010 SE GS-MS instrument manufactured by Shimadzu, Japan. This analytical procedure was meticulously executed in conjunction with the GC-FID thermal program. To verify the identity of FAMEs, the mass fragmentation pattern was compared with authentic standards and reference databases, including NIST08S, NIST08, and Wiley9. Together, this comprehensive methodology ensured the attainment of accurate and reliable results in the process of identifying and characterizing FAMEs within the analyzed samples.

### 2.5. Phytosterols Analysis

The analysis of phytosterols involved silylation and was performed using the QP2010 SE GC–MS instrument manufactured by Shimadzu, Tokyo, Japan. The instrumental conditions are summarized in Table 4 [43,44]. To verify the identity of phytosterols, we compared the mass fragmentation pattern with authentic standards and reference databases, including NIST08S, NIST08, and Wiley9.

### 2.6. Antioxidant Activity

The ABTS^•+^ and DPPH^•^ scavenging activities were assessed following our optimized method [45], based on Thaipong et al. [46]. To measure the ABTS^•+^ scavenging activities, stock solutions of 2.6 mM potassium persulfate solution and 7.4 mM ABTS^+^ solution were prepared, mixed in equal amounts, and were left to react for 12 h at room temperature and in the dark. The solution was then diluted by combining 10 mL of ABTS^•+^ solution with methanol to obtain an absorbance of 1.1 ± 0.02 units at 734 nm, using a spectrophotometer. For each test, a fresh ABTS^•+^ solution was prepared. Extracts (100 µL) were allowed to react with 1900 µL of the ABTS^•+^ solution for 2 h while kept in the dark for the assay. The spectrophotometer was then used to measure the absorbance at 734 nm. Trolox, a water-soluble vitamin E analog, was used as a standard antioxidant reference for comparison among various samples, and the results were expressed in Trolox equivalents (TE).

### 2.7. Statistical Analysis

The flower petal sample from each cultivar underwent a total of four replicate extractions and analyses. A one-way analysis of variance (ANOVA) was conducted using IBM SPSS Statistics (version 28), with a significance level set at 0.05 (Tukey HSD).

## 3. Results and Discussion

### 3.1. Identification of Carotenoids

**Figure 1:**
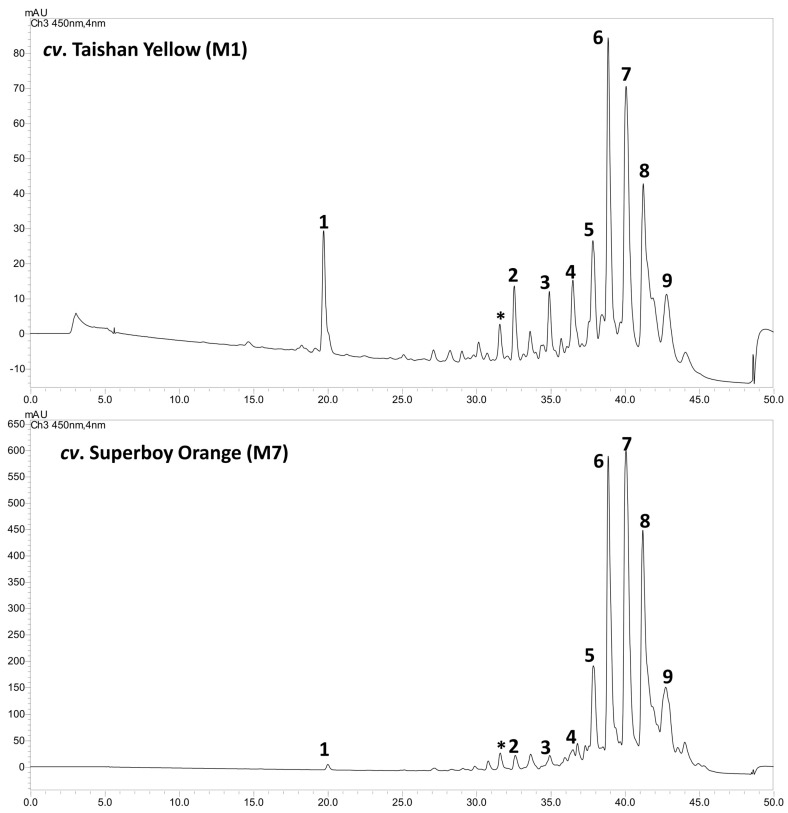
The representative LC-PDA chromatograms (450 nm) of carotenoids identified and quantified from marigold petals. The peak numbers 1–9 correspond to Table 2. * Probably a chlorophyll compound.

**Figure 2:**
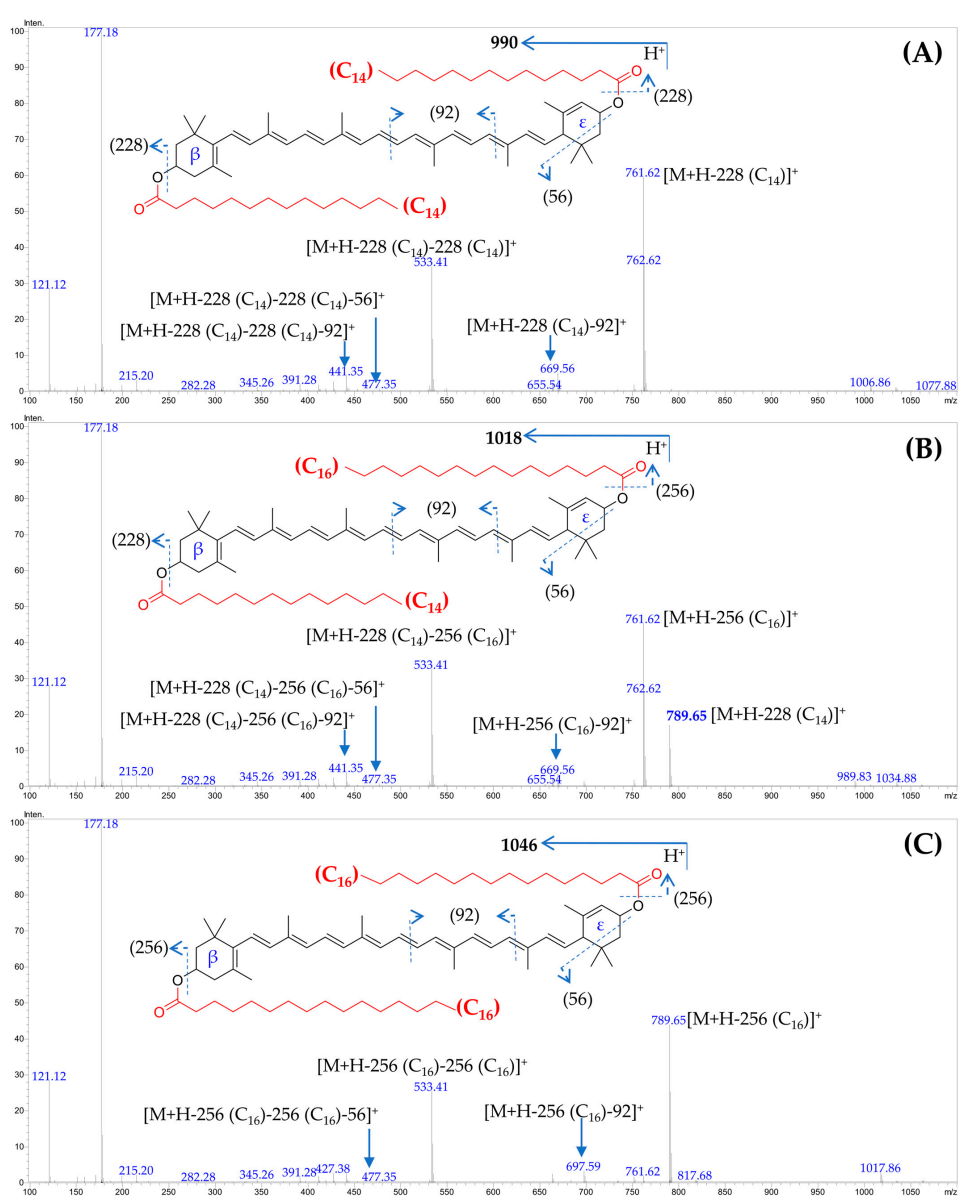
The LC mass spectrum of major lutein esters identified and quantified from marigold petals. (**A**), (all-*E*)-lutein-dimyristate; (**B**), (all-*E*)-lutein-3-*O*- myristate -3′-*O*-palmitate; and (**C**) (all-*E*)-lutein-dipalmitate. C_14_: myristate; C_16_: palmitate.

In the non-saponified lipophylic marigold extract, nine major carotenoids were identified (Table 5, Figure 1). The lutein homodiesters (attachment of the same fatty acid moieties at the β- and ε-rings of the lutein molecule) and heterodiesters (attachment of different fatty acid moieties at the β- and ε-rings of the lutein molecule) were found to dominate in the lipophylic marigold extract of all the studied cultivars. The neutral loss of either one [M+H-FA_1_]^+^ or the other [M+H-FA_2_]^+^, or both the fatty acid moieties [M+H-FA_1_-FA^2^]^+^ were the characteristic fragment ions obtained from these lutein diesters (Table 2, Figure 2). The neutral loss of both fatty acid moieties gives rise to a fragment equivalent to the lutein carbon backbone at m/z 533. Additionally, we observed typical fragmentation patterns of ε-ring-containing structures, including the loss of 56 u due to retro-Diels-Alder fragmentation of the α-ionone moiety and of the carotenoid polyene chain, which involved the elimination of toluene (92 u) (Figure 2) [29,47].

In the case of the lutein heterodiesters, such as peak 7 (Figure 2B), the higher intensity of the fragment ion at m/z 761 [M+H-256 (palmitate)]^+^, compared to m/z 789 [M+H-228 (myristate)]^+^, indicated that palmitic acid is attached at the 3′-*O*′ position of ε-ring, as loss of fatty acids from this position generates a more stable fragment ion than fatty acid acylated to the 3-*O*-position of β-ring [29]. Thus, this heterodiester was assigned as (all-*E*)-lutein-3-*O*-myristate -3′-*O*-palmitate.

### 3.2. Quntititaive Analysis of Carotenoids and Tocopherols

In the present study, nine major carotenoids were identified and quantified from 10 marigold cultivars (Table 6). Among the identified carotenoids, lutein diesters were the most dominating, accounting for 63.76–96.95% (*cv.* Alaska and –*cv.* Durango Orange, respectively) of total carotenoids. Other carotenoids, such as (all-*E*)-lutein, (all-*E*)-β-carotene, (all-*E*)-violaxanthin-3,3′-dimyristate, and (all-*E*)-violaxanthin-3-*O*-myristate-3-*O*′-palmitate were present in a small amount. Among the five major lutein-diesters identified and quantified, (all-*E*)-lutein-3-*O*-myristate-3′-*O*-palmitate was the most dominating carotenoid amond the French (*T. patula* L.)-type cultivars (M5–M10). Conversely, in *cv.* Taishan Orange (M2) and *cv.* Incall Orange (M3; Aztec type; *T. erecta* L.), lutein dipalmitate was the dominant carotenoid.

In the present study, the total lutein contents varied significantly (*p* < 0.05, Tukey HSD) among cultivars, ranging from 23.73–2613.47 µg/g fresh weight (FW) (*cv.* Alaska and *cv.* Superboy Orange, respectively). The significantly highest contents of lutein were recorded in *cv.* Superboy Orange, due to the presence of the highest amount of (all-*E*)-lutein-3-*O*-myristate-3-*O*-laurate (212.13 µg/g FW), (all-*E*)-lutein-dimyristate (602.86 µg/g FW), (all-*E*)-lutein-3-*O*-myristate-3′-*O*-palmitate (753.86 µg/g FW), and (all-*E*)-lutein-3-*O*-stearate-3′-*O*-palmitate (317.97 µg/g FW) (Table 6).

Several studies have attempted to quantify the lutein contents in marigold flower petals. However, most of these studies quantified the lutein contents in saponified samples [26,27,28]. In saponification, fatty acid moieties attached to the carotenoid molecules are removed, yielding free carotenoids. This process makes quantification easy; however, this approach does not provide information about naturally occurring carotenoid esters. In recent years, the availability of high-resolution mass spectrometry techniques has made it possible to separate and identify the complex carotenoid esters in native samples. In one such study, Piccaglia et al. [48] recorded the predominance of lutein dimyristate, lutein myristate palmitate, and lutein dipalmitate in ten types of *T. patula* and *T. erecta*, with a lutein content of 167.7–5699.0 µg/g. Similarly, El-Sayed et al. [20] also recorded the dominance of (all-*E*)-lutein-dimyristate, (all-*E*)-lutein-3-*O*-myristate-3′-*O*-palmitate, and (all-*E*)-lutein-dipalmitate in the seven cultivars of *T. patula* and *T. erecta*, with a lutein content of 167–5752.0 µg/g. The total lutein contents recorded in the present investigation agree with these studies.

This study screened the lipophilic extract of marigold flower petals for tocopherol contents using LC-PDA-MS. Among the studied cultivars, 167.91–338.50 µg/g FW (*cv.* Superboy Yellow and *cv.* Taishan Orange, respectively) of α-tocopherol was recorded (Table 6), while other forms (e.g., δ-, γ-, β-tocopherol) were not found in significant quantities.

To the best of our knowledge, previous studies on the tocopherol contents of marigold flower petals are not available. Gong et al. [49] reported the dominance of α-tocopherol in T. erecta flower petals; however, quantitative analysis was not performed in their study. Among the 62 edible tropical plants screened for α-tocopherol contents, the highest content of 420 µg/g FW was recorded from Sauropus androgynus leaves, while Musa sapientum flower was found to contain 29.8 µg/g FW of α-tocopherol [50]. Considering this, marigold flower petals contain a substantially higher amount of α-tocopherol than commonly consumed herbs. Moreover, α-tocopherol content recorded from marigold flower petals in the present study are substantially higher than the contents recorded in our recent studies from 18 Korean traditional green leafy vegetables (13.6–133.6 µg/g FW) [51], leaf mustard (Brassica juncea (L.) czern.) cultivars (67.16–83.42 µg/g FW) [43], and emerging green leafy vegetables, including Moringa oleifera (22.0–83.7 µg/g FW) [44].

Attributable to the high contents of α-tocopherol in marigold flower petals, their enhanced intake can help to minimize the incidence of oxidative stress-related diseases [36].

### 3.3. Ideal Cultivar for Food Fortification and Commercial Lutein Extraction

Marigold flower petals are the primary source of lutein for commercial extraction [19]. Moreover, marigold flowers can be directly utilized as a food ingredient in fortifying foods with lutein [52]. The present study suggests that among the studied cultivars, flower petals of *cv.* Superboy Orange is the richest source of lutein, which can be utilized in food fortification.

In the present study, the yield of flower petals from the greenhouse experiments was used to calculate the yield on a per hectare (ha) basis. Results showed a flower petal yield of 19.35–47.37 Tons/ha (*cv.* Durango Bee and *cv.* Durango Red, respectively) (Table 7). The previous studies also recorded a similar annual average production (30–60 tons/ha) of *T. erecta* fresh flowers [53].

In the present study, the highest (*p* < 0.05, Tukey HSD) contents of lutein and total carotenoids were obtained from *cv.* Superboy Orange. However, due to a substantially higher flower petal yield with high lutein contents, the *cv.* Durango Red can produce the highest lutein yield (94.45 kg/ha) and total carotenoid yield (97.13 kg/ha). These observations suggest that Durango Red is an ideal candidate for marigold cultivation and could be utilized for commercial lutein extraction.

### 3.4. Phytosterols

**Figure 3:**
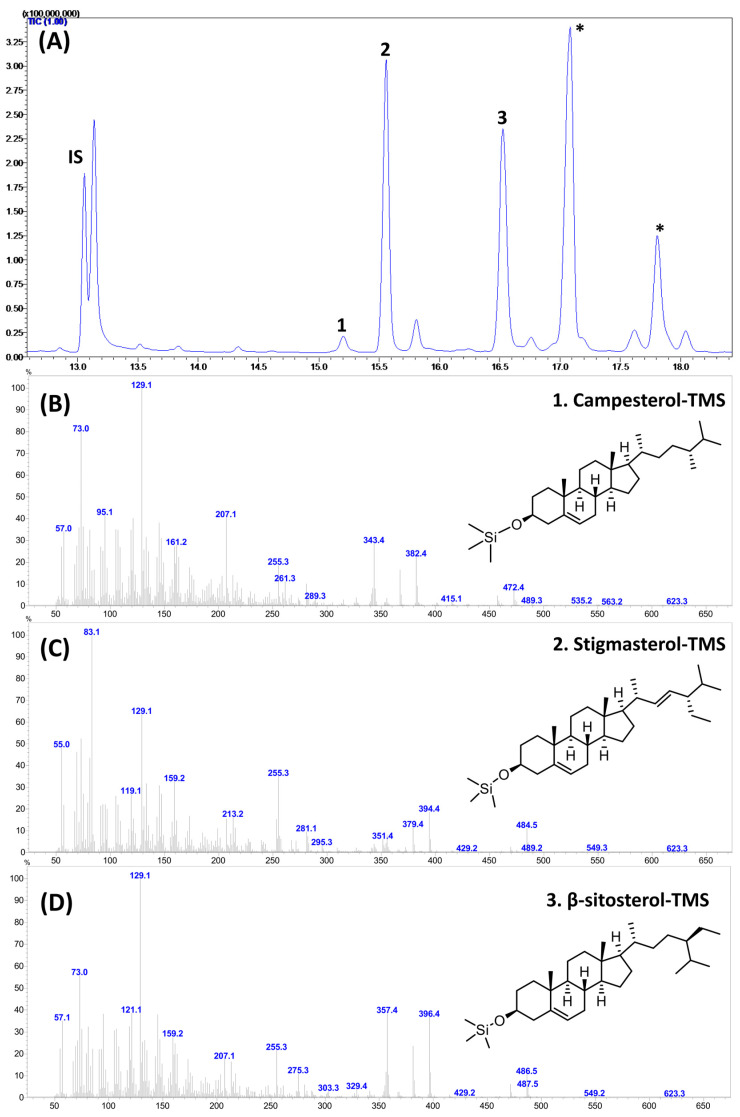
The GC–mass chromatogram (**A**) and mass spectrum of major sterols (**B**–**D**) identified and quantified from the flower petals of various marigold cultivars. IS: internal standard. 1, campesterol; 2, stigmasterol; 3, β-sitosterol. * not a sterol compound.

In the present study, GC–MS analysis of trimethylsiloxy [−O-Si(CH_3_)_3_; TMS] derivatives revealed the presence of campesterol (Campest-5-en-3β-ol), stigmasterol (Stigmasta-5,22-dien-3β-ol), and β-sitosterol (Stigmast-5-en-3β-ol) in the studied samples (Figure 3).

In this study, β-sitosterol was the most prevalent phytosterol among the studied cultivars, ranging between 127.08 (*cv.* Superboy Yellow) and 191.99 µg/g FW (*cv.* Taishan Yellow), which accounted for 46.57–62.43% (*cv.* Taishan Orange and *cv.* Superboy Yellow, respectively) of total phytosterol (Table 8). The highest total sterol content of 333.42 µg/g FW was recorded from *cv.* Taishan Yellow.

To the best of our knowledge, no detailed studies are available on the sterol composition of *Tagetes* spp. However, we have previously recorded the predominance of β-sitosterol in herbs such as red lettuce (73.3 µg/g FW), fenugreek (*Trigonella foenum-graecum* L.; 85.7 µg/g FW), *Moringa oleifera* Lam. (175.9 µg/g FW), *Kaempferia parviflora* Wall. Ex Baker (30.6–36.6 µg/g FW) [54], and *Perilla frutescens* Britt. (27.7–37.9 µg/g FW) [55].

### 3.5. Fatty Acids

In this study, we identified seven prominent fatty acids from the flower petals of ten marigold cultivars, and their relative occurrence (expressed as percentages of the total fatty acids) was determined (Table 9). Among the studied cultivars, palmitic acid (C_16:0_; 33.36–47.43%) was the most dominant fatty acid, followed by linoleic (18:2n6c; 11.30–30.25%) and stearic acid (C_18:0_; 15.99–24.57%). These three fatty acids together accounted for 72.13–86.44% (*cv.* Superboy Orange and *cv.* Taishan Yellow, respectively) of total fatty acids. Moreover, because of the dominance of palmitic and stearic acid, the total saturated fatty acids (SFAs) accounted for 58.17–75.90% (*cv.* Durango Bee and *cv.* Alaska, respectively) of total fatty acids. Interestingly, α-linoleic acid (C_18:3n3_), which commonly occurs in photosynthetic tissues of the plant, was recorded as just 3.55–7.85% (*cv.* Taishan Orange and *cv.* Alaska, respectively) of total fatty acids.

Only a few reports are available on the fatty acid composition of *Tagetes* spp. flowers. Gong et al. [49] reported the dominance of linoleic acid (26.41%), palmitic acid (24.22%), and oleic acid (20.12%) in the *T. erecta* flower lipids extracted utilizing supercritical CO_2_ (SC-CO_2_). Additionally, Zonta et al. [56] reported the dominance of palmitic acid (31.35%), linoleic acid (22.89%), and oleic acid (15.44%) in *T. erecta* flower petals. In agreement with these reports, in the present study, we also recorded the dominance of linoleic acid and palmitic acid; however, we recorded only 1.34–4.57% (*cv.* Taishan Orange and *cv.* Alaska, respectively) of oleic acid. In the present study, a substantial variation recorded for the fatty acid composition among the ten cultivars suggests the existence of significant genetic diversity among the marigold cultivars.

### 3.6. Antioxidant Activities

The lipophylic extract prepared from marigold flower petals showed ABTS^•+^ and DPPH^•^ radical scavenging potential (Table 10). In the present study, the extract prepared from *cv.* Taishan Orange, Superboy Orange, and Durango Red presented the highest (*p* < 0.05, Tukey HSD) ABTS^•+^ scavenging activities of 402.33, 400.10, and 389.30 mg TE/100g FW, respectively. Conversely, *cv.* Durango Orange, Taishan Orange, Superboy Orange, and Incall Orange exhibited the highest DPPH^•^ radical scavenging activities of 241.19, 250.82, 253.22, and 258.64 mg TE/100 g FW, respectively.

The ABTS^•+^ and DPPH^•^ radical scavenging assays used in this study are based on the electron transfer (ET) mechanism. Consistent with earlier findings [46,57], a strong correlation (R = 0.787) was observed between these assays (Table 8).

Carotenoids, tocopherols, ascorbic acid, and phenolic compounds (flavonoids and phenolic acids) are the principal antioxidants found in herbs [58]. The present study reported a high correlation coefficient (R) of 0.803 and 0.686 between the total lutein contents and DPPH^•^ and ABTS^•+^ radical scavenging activities, respectively (Table 11). Gong et al. [49] also reported a similar correlation coefficient between the total lutein contents of marigold flowers and DPPH^•^ and ABTS^•+^ radical scavenging activities. Moreover, the present study reported a high correlation coefficient of 0.732 between the α-tocopherol contents and DPPH^•^ radical scavenging activities. These observations suggest that the radical scavenging activities of the marigold flower extracts are related to their lutein and α-tocopherol content.

### 3.7. Potential Impacts and Applications of the Obtained Results

Lutein is known for its potential health benefits, particularly in supporting eye health and acting as an antioxidant; the high lutein content with high antioxidant potential in marigold flower petals, especially *cv.* Superboy Orange, suggests an excellent candidate for incorporation into functional foods and nutraceuticals. Our findings regarding the cultivars with the highest lutein content can guide the development of lutein-enriched functional foods.

This study emphasizes the importance of maximizing lutein yield from marigold cultivation for cost-efficient extraction. Manufacturers can apply this insight to cultivate the high flower-yielding and lutein-rich cultivars, such as *cv.* Durango Red. This could lead to cost-effective production of lutein for various applications. Moreover, this study revealed that the contents of nutritionally vital constituents varied significantly among the marigold cultivars. These findings provide a strong foundation for further exploration of the genetic potential of marigolds to identify nutrient-rich cultivars for food fortification and commercial lutein extraction.

### 3.8. Limitations of the Study

In this study, ten cultivars of marigold commonly cultivated in Korea were grown under greenhouse conditions and utilized for the quantitative assessment of lutein-esters and other nutritionally vital constituents. Among the studied cultivars, *cv.* Durango Red produced the highest flower yield with high contents of lutein. These results suggest that *cv.* Durango Red is an ideal candidate for marigold cultivation and could be utilized for commercial lutein extraction. However, for commercial lutein extraction, marigolds are cultivated in the field. Under field conditions, the studied cultivars’ yield performance and lutein contents may change. Thus, field studies are warranted to establish the results obtained in the present study.

## 4. Conclusions

The commercial significance of lutein sourced from marigold flower petals is profound due to its versatile applications in functional food, feed, cosmetics, and pharmaceuticals. Significant differences in carotenoids (including lutein), α-tocopherol, phytosterols, fatty acid contents, and antioxidant activities were observed among the cultivars. The highest contents of total carotenoids (2723.11 µg/g FW) and lutein (2613.47 µg/g FW) were found in *cv*. Superboy Orange. The predominant fatty acids were palmitic acid (33.36–47.43%), followed by linoleic (11.30–30.25%) and stearic acid (15.99–24.57%). The highest level of total phytosterols (362.51 µg/g FW) was present in *cv*. Taishan Orange.

In addition to the high lutein contents, maximizing the lutein yield from the unit area of marigold cultivation holds paramount importance in the cost-efficient extraction of lutein. The present study suggests that due to the substantially higher flower petal yield with high lutein contents of 1995.90 µg/g FW, the *cv*. Durango Red can produce the highest lutein yield of 94.45 kg/ha. These observations suggest that *cv*. Durango Red is an ideal candidate for marigold cultivation and could be utilized for commercial lutein extraction. Moreover, *cv*. Superboy Orange is the richest source of lutein with the highest antioxidant potential, which can be used in functional food preparations.

## Figures and Tables

**Table 1:** List of marigold cultivars investigated in the present study.

Cultivar No.	Cultivar Type	Botanical Name	Cultivar (*cv*.)	Flower Color	Picture
M1	Aztec	*Tagetes erecta* L.	Taishan Yellow	Yellow	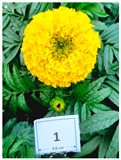
M2	Aztec	*Tagetes erecta* L.	Taishan Orange	Orange	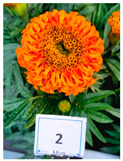
M3	Aztec	*Tagetes erecta* L.	Incall Orange	Orange	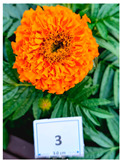
M4	Aztec	*Tagetes erecta* L.	Alaska	Yellow	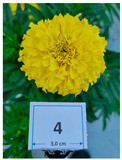
M5	French	*Tagetes patula* L.	Durango Yellow	Yellow	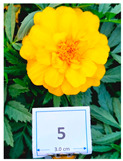
M6	French	*Tagetes patula* L.	Superboy Yellow	Yellow	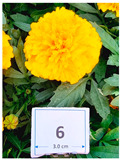
M7	French	*Tagetes patula* L.	Superboy Orange	Orange	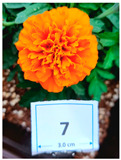
M8	French	*Tagetes patula* L.	Durango Orange	Orange	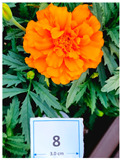
M9	French	*Tagetes patula* L.	Durango Red	Red	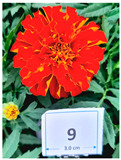
M10	French	*Tagetes patula* L.	Durango Bee	Yellow	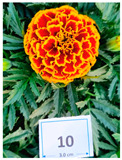

**Table 2:** LC-PDA-MS parameters utilized for the analysis of carotenoids and tocopherols.

**Liquid Chromatograph (LC)**	
Column	YMC C30 carotenoid column (150 mm × 4.6 mm, 3 μm; YMC, Wilmington, NC, USA)
Column oven temperature	20 °C
Mobile phase	A—Mixture of 95/5 methanol and water (*v*/*v*) that contains 5 mM ammonium formate * B—Mixture of 90/7/3 methyl tertiary butyl ether/methanol/water (*v*/*v*/*v*), containing 5 mM ammonium formate *
Injection volume	2 µL
Flow rate	0.5 mL/min
Gradient program	0% B to 100% B in 45 min; 5-min post-run at 0% B
**Mass Spectrometer (MS)**	
MS program	0.0 min—diverter valve to drain 8.0 min—diverter valve to MS
Ionization method	Atmospheric-pressure chemical ionization (APCI) in positive mode
Interface temperature	400 °C
Corona needle voltage	4.0 kv
DL temperature	300 °C
Heat block temperature	300 °C
Drying gas flow	10 L/min
Nebulizing gas flow	3 L/min
Data acquisition	1.86 Hz
Q1 resolution	±20 ppm

* Ammonium formate was added to enhance the ionization [42].

**Table 3:** Gas chromatography (GC)–flame ionization detection (FID) parameters used for the analysis of fatty acid methyl esters (FAMEs).

Parameters	Value		
Injection volume	1 µL		
Injection mode	Split, 5:1		
Split flow	10 mL/min		
Injection temperature	250 °C		
Septum purge flow	3 mL/min		
Inlet total flow	15 mL/min		
Carrier gas flow	2 mL/min (Nitrogen)		
Inlet pressure	54.901 psi		
Detector H_2_ flow	30 mL/min		
Detector makeup flow	25 mL/min (Nitrogen)		
Detector airflow	400 mL/min		
Column	SP-2560 capillary with a length of 100 m, a film thickness of 0.20 µm, and an inner diameter of 0.25 mm, sourced from Merck KgaA in Darmstadt, Germany.		
Column oven program	Rate	Final temperature	Temperature hold time
	-	140 °C	1 min
	5 °C/min	240 °C	15 min
FID detector temperature	260 °C		
Total run time	45 min		
Post run time	5 min		

**Table 4:** Gas chromatography (GC)–mass spectrometry (MS) parameters used for the analysis of phytosterols.

**Gas Chromatograph (GC)**			
Injection mode	Split		
Carrier gas	Helium		
Injection temperature	260 °C		
Column oven temperature	150 °C		
Flow control mode	Liner velocity		
Total flow	8.6 mL/min		
Column flow	0.93 mL/min		
Pressure	86.5 kPa		
Purge flow	3.0 mL/min		
Liner velocity	36.7 cm/s		
Column	DB-5 ms with 30 m length, 0.25 μm film thickness, 0.25 mm ID sourced from Agilent Technologies Canada, Inc.		
Column oven program	Rate	Final temperature (°C)	Temperature hold time
	-	150 °C	1 min
	20 °C/min	300 °C	30 min
Total program time	38.5 min		
**Mass Spectrometer (MS)**			
Interface temperature	280 °C		
Ion source temperature	260 °C		
Solvent cut time	3 min		
Acquiring mode	Scan		
Start and End *m*/*z*	50.00 to 650.00		
Start and end time	6 to 38 min		
Scan speed	2500		

**Table 5:** The UV–VIS characteristics and mass spectrometry of carotenoids identified and quantified from marigold petals.

Peak ^1^	Carotenoid	RT (min) ^2^	λ_max_ (nm) ^3^	[M+H]^+^ (*m*/*z*)	Other Characteristic Fragment Ions (*m*/*z*)
1	(all-*E*)-lutein	19.95	447, 471	nd	551.42 [M+H-18]^+^
2	(all-*E*)-β-carotene	32.60	451, 476	537.44	440.40
3	(all-*E*)-violaxanthin-3,3′-dimyristate	34.92	419, 441, 470	1021.81	1003.81 [M+H-18]^+^, 929.75 [M+H-92]^+^, 793.61 [M+H-228 (C_14_)]^+^, 547.39 [M+H-18-228 (C_14_)-228 (C_14_)]^+^
4	(all-*E*)-violaxanthin-3*-O-*myristate-3-*O*′-palmitate	36.50	419, 441, 470	1049.85	793.61 [M+H-256 (C_16_)]^+^, 547.39 [M+H-18-228 (C_14_)-256 (C_16_)]^+^
5	(all-*E*)-lutein- 3*-O-* myristate-3*-O-*laurate	37.86	445, 473	nd	762.62 [M+H-200 (C_14_)]^+^, 733.59 [M+H-228 (C_12_)]^+^, 641.53 [M+H-228 (C_14_)-92]^+^, 533.41 [M+H-200 (C_12_)-228 (C_14_)]^+^
6	(all-*E*)-lutein-dimyristate	38.88	446, 473	nd	761.62 [M+H-228 (C_14_)]^+^, 669.56 [M+H-228 (C_14_)-92]^+^, 533.41 [M+H-228 (C_14_)-228 (C_14_)]^+^, 477.35 [M+H-228 (C_14_)-228 (C_14_)-56]^+^, 441.35 [M+H-228 (C_14_)-228 (C_14_)-92]^+^
7	(all-*E*)-lutein-3*-O-* myristate -3′*-O-*palmitate	40.08	446, 473	nd	789.62 [M+H-228 (C_14_)]^+^, 761.62 [M+H-256 (C_16_)]^+^, 669.56 [M+H-256 (C_16_)-92]^+^, 533.41 [M+H-228 (C_14_)-256 (C_16_)]^+^, 477.35 [M+H-228 (C_14_)-256 (C_16_)-56]^+^, 441.35 [M+H-228 (C_14_)-256 (C_16_)-92]^+^
8	(all-*E*)-lutein-dipalmitate	41.42	446, 473	nd	789.65 [M+H-256 (C_16_)]^+^, 697.59 [M+H-256 (C_16_)-92]^+^, 533.41 [M+H-256 (C_16_)-256 (C_16_)]^+^, 441.35 [M+H-256 (C16)-256 (C_16_)-92]^+^
9	(all-*E*)-lutein-3*-O-* stearate-3′*-O-*palmitate	42.78	446, 473	nd	817.68 [M+H-256 (C_16_)]^+^, 789.65 [M+H-284 (C_18_)]^+^, 725.62 [M+H-256 (C_16_)-92]^+^, 697.58 [M+H-284 (C_18_)-92]^+^, 533.41 [M+H-256 (C_16_)-284 (C_18_)]^+^, 477.35 [M+H-256 (C_16_)-284 (C_18_)-56]^+^, 441.34 [M+H-256 (C_16_)-284 (C_18_)-92]^+^

C_18_: stearate; C_16_: palmitate; C_14_: myristate; C_12_: laurate; nd: not detected. ^1^ Peak numbers 1–9 correspond to Figure 1. ^2^ Retention time (RT) observed in LC-PDA-MS analysis, an average of all the sample runs. ^3^ Recorded by PDA detector during LC-PDA-MS analysis.

**Table 6:** α-tocopherol and carotenoid contents of flower petals of various marigold cultivars.

	M1	M2	M3	M4	M5	M6	M7	M8	M9	M10
α-tocopherol	321.10 ± 8.27	338.50 ± 1.42 ^a^	317.27 ± 8.11	221.83 ± 8.74	252.57 ± 10.82	167.91 ± 16.68 ^b^	271.85 ± 8.29	305.09 ± 23.09	270.71 ± 11.32	306.86 ± 13.01
(all-*E*)-lutein	6.22 ± 1.05 ^b^	9.83 ± 0.08	7.83 ± 1.17	7.40 ± 1.52 ^b^	6.47 ± 0.63 ^b^	5.91 ± 0.85 ^b^	6.95 ± 0.87 ^b^	6.76 ± 0.01 ^b^	7.82 ± 0.34	13.41 ± 1.75 ^a^
(all-*E*)-β-carotene	1.05 ± 0.22 ^b^	5.84 ± 0.25	5.74 ± 0.26	0.53 ± 0.35 ^b^	2.97 ± 0.29	2.42 ± 0.45	15.28 ± 0.60 ^a^	8.45 ± 1.17	6.31 ± 0.28	3.31 ± 0.01
(all-*E*)-violaxanthin-3,3′-dimyristate	2.94 ± 0.20 ^b^	23.59 ± 0.87	21.31 ± 0.55	0.93 ± 0.09 ^b^	6.27 ± 0.05	5.27 ± 0.69	44.62 ± 2.13 ^a^	24.13 ± 4.06	23.65 ± 1.24	6.52 ± 0.59
(all-*E*)-violaxanthin-3*-O-*myristate-3-*O*′-palmitate	4.63 ± 0.28	28.78 ± 0.61	26.56 ± 0.50	0.43 ± 0.05 ^b^	5.61 ± 0.07	3.92 ± 0.08	49.74 ± 1.92 ^a^	28.63 ± 4.65	26.82 ± 1.89	6.89 ± 0.67
(all-*E*)-lutein-3*-O-*myristate-3*-O-*laurate	6.34 ± 0.33 ^b^	98.91 ± 2.48	91.11 ± 0.27	2.22 ± 0.02 ^b^	35.10 ± 3.11	18.28 ± 0.14	212.13 ± 5.13 ^a^	155.97 ± 13.70	162.19 ± 13.54	43.74 ± 2.56
(all-*E*)-lutein-dimyristate	16.33 ± 0.78 ^b^	298.50 ± 7.22	269.27 ± 3.69	4.98 ± 0.18 ^b^	70.08 ± 6.45	41.76 ± 2.70	602.86 ± 15.94 ^a^	409.93 ± 22.47	415.79 ± 27.72	102.08 ± 6.39
(all-*E*)-lutein-3*-O-*myristate -3′*-O-*palmitate	19.46 ± 1.00 ^b^	594.78 ± 14.20	543.05 ± 9.23	4.95 ± 0.69 ^b^	77.69 ± 3.38	55.37 ± 1.74	753.86 ± 15.29 ^a^	676.20 ± 29.20	653.03 ± 32.84	109.54 ± 9.98
(all-*E*)-lutein-dipalmitate	13.62 ± 0.74 ^b^	747.43 ± 18.37 ^a^	701.72 ± 12.86	2.88 ± 0.53 ^b^	56.28 ± 0.64	41.02 ± 2.15	719.69 ± 16.32	666.48 ± 18.84	595.96 ± 30.71	69.80 ± 8.02
(all-*E*)-lutein-3*-O-*stearate-3′*-O-*palmitate	6.24 ± 0.40 ^b^	269.24 ± 4.56	267.10 ± 4.89	1.31 ± 0.33 ^b^	39.48 ± 1.41	28.36 ± 1.07	317.97 ± 3.72 ^a^	252.03 ± 24.76	161.11 ± 14.94	43.71 ± 3.68
Total lutein	68.20 ± 4.02 ^b^	2018.69 ± 46.75	1880.08 ± 32.11	23.73 ± 1.82 ^b^	285.09 ± 11.52	190.70 ± 1.26	2613.47 ± 57.28 ^a^	2167.38 ± 108.95	1995.90 ± 120.09	382.27 ± 32.38
Total carotenoids	76.82 ± 4.32 ^b^	2076.90 ± 48.48	1933.70 ± 33.41	25.62 ± 1.94 ^b^	299.95 ± 11.78	202.31 ± 0.05	2723.11 ± 61.93 ^a^	2228.58 ± 118.83	2052.68 ± 123.51	398.99 ± 33.63

Values (µg/g FW) are the mean ± standard deviation of four determinations. The superscript letters “a” and “b” indicate the highest and lowest statistically significant values among the different cultivars (*p* < 0.05, Tukey HSD). The sample numbers M1–M10 correspond to Table 1. Laurate: C_12:0_; myristate C_14:0_; palmitate: C_16:0_; stearate: C_18:0_.

**Table 7:** The estimates of flower petals, lutein, and total carotenoid yield per hectare (ha) basis.

Cultivar Number	Flower Petals Yield (Tons/ha)	Lutein Yield (kg/ha)	Carotenoid Yield (kg/ha)
M1	43.66 ± 5.04 ^a^	2.99 ± 0.52 ^b^	3.37 ± 0.57 ^b^
M2	24.26 ± 4.20	49.12 ± 9.62	50.54 ± 9.90
M3	37.69 ± 7.84	70.67 ± 13.53 ^b^	72.68 ± 13.90
M4	30.06 ± 2.87	0.71 ± 0.03 ^b^	0.77 ± 0.44 ^b^
M5	28.09 ± 3.39	7.99 ± 0.83	8.41 ± 0.88 ^b^
M6	31.76 ± 3.98	6.06 ± 0.74 ^b^	6.43 ± 0.80 ^b^
M7	32.37 ± 3.82	84.76 ± 11.81	88.32 ± 12.38
M8	30.06 ± 4.61	64.82 ± 7.05	66.62 ± 7.07
M9	47.37 ± 4.34 ^a^	94.45 ± 8.69 ^a^	97.13 ± 8.93 ^a^
M10	19.35 ± 6.27 ^b^	7.55 ± 3.02 ^b^	7.55 ± 3.02 ^b^

Values are mean ± standard deviation of four determinations. The superscript letters “a” and “b” indicate the highest and lowest statistically significant values among the different cultivars (*p* < 0.05, Tukey HSD). The sample numbers M1–10 correspond to Table 1.

**Table 8:** Phytosterol composition of flower petals from various marigold cultivars.

Cultivar Number	Campesterol	Stigmasterol	β-Sitosterol	Total Sterol
M1	13.11 ± 0.67	128.32 ± 3.49	191.99 ± 6.92 ^a^	333.42 ± 9.74
M2	10.57 ± 0.76 ^b^	183.12 ± 3.63 ^a^	168.81 ± 0.11	362.51 ± 4.29 ^a^
M3	10.36 ± 0.70 ^b^	157.34 ± 1.30	163.09 ± 4.59	330.79 ± 5.19
M4	21.65 ± 1.35	103.19 ± 3.71	149.04 ± 2.99	273.88 ± 0.63
M5	32.88 ± 0.27 ^a^	96.24 ± 0.47	134.04 ± 11.49 ^b^	263.16 ± 10.75
M6	13.17 ± 0.29	63.31 ± 5.90 ^b^	127.08 ± 6.41 ^b^	203.55 ± 12.01 ^b^
M7	25.87 ± 0.74	91.38 ± 1.01	171.87 ± 0.25	289.12 ± 1.50
M8	29.27 ± 1.72	92.95 ± 3.30	135.46 ± 6.31 ^b^	257.68 ± 4.73
M9	26.86 ± 0.84	71.71 ± 5.19	132.62 ± 1.80 ^b^	231.19 ± 2.55
M10	27.82 ± 0.98	91.73 ± 3.68	138.14 ± 7.29 ^b^	257.70 ± 4.59

Values (µg/g FW) are the mean ± standard deviation of four determinations. The superscript letters “a” and “b” indicate the highest and lowest statistically significant values among the different cultivars (*p* < 0.05, Tukey HSD). The cultivar numbers M1–10 correspond to Table 1.

**Table 9:** Fatty acid composition of flower petals from various marigold cultivars.

Peak Number	FAME	RT (min)	M1	M2	M3	M4	M5	M6	M7	M8	M9	M10
1	C_12:0_ (Lauric)	13.81	nd ^b^	1.58 ± 0.06 ^a^	0.84 ± 0.32	nd ^b^	0.34 ± 0.09 ^b^	nd^b^	1.90 ± 0.32 a	1.41 ± 0.58 ^a^	1.69 ± 0.23 ^a^	0.19 ± 0.07 ^b^
2	C_14:0_ (Myristic)	17.18	4.05 ± 1.73 ^b^	17.32 ± 0.57 ^a^	11.20 ± 2.35	5.56 ± 0.37 ^b^	6.32 ± 0.97 ^b^	4.97 ± 0.19 ^b^	17.39 ± 1.11 ^a^	15.12 ± 2.64 ^a^	15.25 ± 1.10 ^a^	5.44 ± 0.64 ^b^
3	C_16:0_ (Palmitic)	20.89	35.12 ± 3.09	47.43 ± 0.34 ^a^	44.04 ± 0.48	34.86 ± 1.85	33.36 ± 0.31 ^b^	33.58 ± 1.29	36.74 ± 1.05	38.79 ± 1.07	40.47 ± 1.42	32.22 ± 1.66 ^b^
4	C_18:0_ (Stearic)	24.52	21.07 ± 0.24	17.48 ± 0.53	19.82 ± 1.59	22.61 ± 0.84	22.21 ± 0.27	24.57 ± 1.17 ^a^	19.29 ± 0.86	18.77 ± 0.29	15.99 ± 0.10 ^b^	20.31 ± 1.80
5	C_18:1n9c_ (Oleic)	25.61	2.24 ± 0.49	1.34 ± 0.01 ^b^	1.86 ± 0.28 ^b^	2.79 ± 0.38	3.10 ± 0.21	3.27 ± 1.04	2.73 ± 1.09	2.11 ± 0.34	2.52 ± 0.06	4.57 ± 2.94 ^a^
6	C_18:2n6c_ (Linoleic)	27.24	30.25 ± 3.89 ^a^	11.30 ± 1.20 ^b^	17.22 ± 1.20	26.72 ± 2.19 ^a^	28.57 ± 0.62 ^a^	27.06 ± 1.56 ^a^	16.11 ± 1.49	18.49 ± 4.45	18.62 ± 2.26	29.42 ± 1.85 ^a^
7	C_18:3n3_ (α-Linolenic)	29.09	7.26 ± 0.69	3.55 ± 0.30 ^b^	5.03 ± 0.65	7.47 ± 0.49	6.09 ± 0.62	6.56 ± 0.05	5.85 ± 0.77	5.32 ± 0.47	5.46 ± 0.45	7.85 ± 0.61 ^a^
	∑SFAs		60.24 ± 5.07 ^b^	83.81 ± 1.50 ^a^	75.90 ± 1.56	63.02 ± 3.06 ^b^	62.23 ± 1.02 ^b^	63.12 ± 2.65 ^b^	75.31 ± 3.35	74.09 ± 4.58	73.40 ± 2.65	58.17 ± 4.18 ^b^
	∑PUFAs		37.52 ± 4.58 ^a^	14.85 ± 1.50 ^b^	22.24 ± 1.85	34.19 ± 2.68 ^a^	34.67 ± 1.24 ^a^	33.61 ± 1.61 ^a^	21.96 ± 2.26	23.81 ± 4.92	24.08 ± 2.71	37.27 ± 1.23 ^a^
	∑PUFAs/∑SFAs		0.63 ± 0.13 ^a^	0.18 ± 0.02 ^b^	0.29 ± 0.03	0.54 ± 0.07 ^a^	0.56 ± 0.03 ^a^	0.53 ± 0.05 ^a^	0.29 ± 0.04	0.32 ± 0.09	0.33 ± 0.05	0.64 ± 0.07 ^a^

Values (mean ± standard deviation) are % of the total fatty acids methyl esters (FAMEs) from four determinations. The superscript letters “a” and “b” indicate the highest and lowest statistically significant values among the different cultivars (*p* < 0.05, Tukey HSD). The cultivar numbers M1–10 correspond to Table 1. RT: retention time; FAME: fatty acid methyl ester; SFAs: total saturated fatty acids; PUFAs: total polyunsaturated fatty acids; nd: not detected.

**Table 10:** The antioxidant activity of flower petals from various marigold cultivars.

Cultivar Number	Antioxidant Assay	
	ABTS^•+^ Activity	DPPH^•^ Activity
M1	334.53 ± 23.23	199.69 ± 12.50
M2	402.33 ± 15.49 ^a^	250.82 ± 2.08 ^a^
M3	347.94 ± 12.90	258.64 ± 1.39 ^a^
M4	342.36 ± 15.06	199.09 ± 13.20
M5	351.30 ± 13.34	164.20 ± 11.81
M6	237.30 ± 7.31 ^b^	114.28 ± 6.95
M7	400.10 ± 12.04 ^a^	253.22 ± 4.86 ^a^
M8	353.16 ± 10.32	241.19 ± 6.25 ^a^
M9	389.30 ± 3.87 ^a^	217.74 ± 2.78
M10	259.28 ± 7.74 ^b^	181.05 ± 7.64

Values (mg of Trolox equivalent/100 g FW) are four replicates’ mean ± standard deviation. The superscript letters “a” and “b” indicate the highest and lowest statistically significant values among the different cultivars (*p* < 0.05, Tukey HSD). The cultivar numbers M1–10 correspond to Table 1.

**Table 11:** The correlation coefficient (*R*) between α-tocopherol, total lutein, and phytosterol contents, and the antioxidant potential of flower petals of various marigold cultivars.

	*α-Tocopherol*	*Total Phytosterol*	*Total Lutein*	*ABTS^•+^ Activity*	*DPPH^•^ Activity*
α-Tocopherol	1.000				
Total phytosterol	0.755	1.000			
Total lutein	0.438	0.241	1.000		
ABTS^•+^ activity	0.464	0.513	0.686	1.000	
DPPH^•^ activity	0.732	0.654	0.803	0.787	1.000

## Data Availability

Data are contained within the article.

## Supplementary Materials

The following supporting information can be downloaded at https://www.mdpi.com/article/10.3390/foods12193549/s1; Figure S1: Outline of the method used for extraction of lipophilic compounds containing carotenoids, sterols, and fatty acids; Figure S2: Outline of methods used for the hydrolysis and fatty acid methyl preparation (FAME) for GC–FID and GC–MS analysis; Figure S3: Outline of methods used for the hydrolysis and silylation of sterols for GC–MS.
