# Supplementary material for: Quantitative Profiling of Carotenoids, Tocopherols, Phytosterols, and Fatty Acids in the Flower Petals of Ten Marigold (Tagetes spp. L.) Cultivars
Source: Foods. 2023 Sep 24;12(19):3549. doi: 10.3390/foods12193549 (PMC10572322; doi:10.3390/foods12193549)
Supplement: Supplementary file 1 [file foods-12-03549-s001.zip › foods-2583834-supplementary.pdf]

Figure S1. Outline of the method used for extraction of lipophilic compounds containing carotenoids, sterols, and fatty acids

2g fresh marigold flower petals in 50 ml falcon tube

↓

Samples were homogenized with 25 ml (acetone/ethanol/cyclohexane, 1:1:2, v/v)

↓

Bath sonication (JAC-2010; 300 w and 60 Hz) for 10 min, followed by ultra-shaking for 2 min in collomix viba x.30 (Tinting Solutions B.V., Nederland) for the efficient disintegration and complete extraction

↓

Vacuum filtered, and pellets were extracted again with 20 ml extraction solvent

↓

The filtrate containing lipophilic compounds was pooled, transferred to a 250 ml round-bottom flask (Duran, Wertheim, Germany), and vacuum-dried in a rotary evaporator at 35 °C

↓

Lipophilic compounds were recovered in 4 ml hexane and transferred to a 5 ml glass vial fitted with PTFE faced rubber-lined cap. These crude lipids were utilized as follows:

Lipophilic compounds in 4 ml hexane

For carotenoids and tocols analysis by LC-PDA-MS, one ml of the crude lipids sample was filtered through a 0.45 μm syringe filter and transferred to a 1.5 ml autosampler vial

0.5 ml of sample was hydrolyzed, converted to fatty acid methyl esters (FAMEs) and analyzed by GC-Flame ionization detection (FID) and GC-MS.

0.5 ml of the sample was hydrolyzed with methanolic KOH, silylated, and utilized for sterols analysis by gas chromatograph-mass spectrometry (GC-MS).

Figure S2. Outline of methods used for the hydrolysis and fatty acid methyl preparation (FAME) for GC-FID and GC-MS analysis

0.5 ml of crude lipids sample was transferred into a 5 ml glass vial fitted with PTFE-faced rubber lined cap

↓

The solvent was evaporated to dryness under a gentle stream of nitrogen

↓

1 ml of 0.5 M methanolic potassium hydroxide (KOH) was added and incubated at 85 °C for 30 min (for hydrolysis) in a heat block

↓

After cooling, and 1 ml of 1 M NaCl and 2 ml of hexane were added for partitioning

↓

One ml of upper clear hexane phase was carefully transferred into a new 5 ml glass vial fitted with PTFE faced rubber lined cap

↓

The solvent was evaporated to dryness under a gentle stream of nitrogen

↓

2 ml methanolic-HCl (5% acetyl chloride in methanol) was added and incubated for 1h at 60 ° in a heat block

↓

After cooling, 2 ml hexane and 1ml 1M NaCl was added

↓

1 ml upper hexane fraction of recovered, filtered through a 0.45 μm PTFE syringe filter packed with a pinch of anhydrous sodium sulfate (to absorb the traces of water) and transferred to 1.5 ml autosampler vial for GC-FID and GC-MS analysis

Figure S3. Outline of methods used for the hydrolysis and silylation of sterols for GC-MS

0.5 ml of sample containing lipophilic compounds was transferred into a 5 ml glass vial fitted with PTFE faced rubber-lined cap

↓

10 μg of 5β-cholestan-3α-ol (internal standard) was added

↓

The solvent was evaporated to dryness under a gentle stream of nitrogen

↓

1 ml of 0.5 M methanolic KOH was added, and the mixture was heated to 85 °C in a water bath for 30 minutes (for hydrolysis)

↓

Samples were immediately cooled, and 1 ml of 1 M NaCl and 2 ml of hexane were added for partitioning

↓

One ml of upper clear hexane phase containing phytosterols was carefully transferred into a new 5 ml glass vial fitted with PTFE faced rubber lined cap

↓

The solvent was evaporated to dryness under a gentle stream of nitrogen

↓

For silylation of phytosterols, 50 μl of N, O-bis(trimethylsilyl)trifluoroacetamide (BSTFA) containing 1% trimethylchlorosilane (TMCS) and 1 ml pyridine were added and incubated at 60 °C for 60 min

↓

Contents were filtered through a 0.45 μm PTFE syringe filtered and transferred to a 1.5 ml autosampler vial for GC-MS analysis
